# Structural variation in humans and our primate kin in the era of telomere-to-telomere genomes and pangenomics

**DOI:** 10.1016/j.gde.2024.102233

**Published:** 2024-07-23

**Authors:** Joana L Rocha, Runyang N Lou, Peter H Sudmant

**Affiliations:** 1 Department of Integrative Biology, University of California, Berkeley, Berkeley, USA; 2 Center for Computational Biology, University of California, Berkeley, Berkeley, USA

## Abstract

Structural variants (SVs) account for the majority of base pair differences both within and between primate species. However, our understanding of inter- and intra-species SV has been historically hampered by the quality of draft primate genomes and the absence of genome resources for key taxa. Recently, advances in long-read sequencing and genome assembly have begun to radically reshape our understanding of SVs. Two landmark achievements include the publication of a human telomere-to-telomere (T2T) genome as well as the development of the first human pangenome reference. In this review, we first look back to the major works laying the foundation for these projects. We then examine the ways in which T2T genome assemblies and pangenomes are transforming our understanding of and approach to primate SV. Finally, we discuss what the future of primate SV research may look like in the era of T2T genomes and pangenomics.

## Comparative genomics of structural variation among primates in the pre-telomere-to-telomere era

In the wake of the human genome project [1], several draft primate genomes were sequenced and assembled [1–5]. While these references provided myriad novel insights into recent evolution in the primate lineage, comparative analyses of structural variation were challenging to perform for these species due to the low contiguity and completeness of these resources. Enormous gains were made improving the contiguity of several of these assemblies (gorilla, chimpanzee, orangutan, bonobo, and macaque) [6–8] using multiplatform approaches combining early PacBIO RS II with Illumina short-read sequencing, Bionano optical mapping, and Fluorescence in situ hybridization and Bacterial Artificial Chromosome clone sequencing (see Ref. [9] for a detailed perspective). These improved genomes provided the first glimpses of shared and species-specific primate structural variation across vastly different evolutionary and physical scales, including hundreds of megabases of lineage-specific segmental duplications (SDs). Furthermore, these data enabled the discovery of thousands of functionally relevant human-specific structural variants (SVs) located nearby genes involved in brain function, which were supported by cell type–specific expression differences. Though still technically ‘draft references’, these sequencing efforts demonstrated not only the extensive interspecies diversity of SVs in primates but also the molecular and phenotypic consequences of this genetic variation.

Inversions were one class of SV, however (among several others, see below), which remained challenging to resolve even in these improved assemblies because of their balanced nature and their placement in complex repetitive sequences. Employing Strand-seq [10], in which the Watson and Crick strands of DNA are independently labeled, Porubsky et al. were able to resolve and map breakpoints of more than 1000 inversions across great apes [11]. Remarkably, 23 loci exhibited inversions that are ‘toggling’, that is, recurrently flipping, among great ape species over the last 15 million years (MYA). Large inversions also tended to impact the expression of nearby genes and were enriched almost 2.5-fold on the X-chromosome. Strand-seq was also employed to identify inversions in gibbons [12]. Gibbons exhibited fivefold as many lineage-specific inversions as other primates with only a small fraction driven by nonallelic homologous recombination, in contrast to many of the recurrent events identified by Porubsky. Furthermore, many of these inversions were heterozygous, indicating a high level of inversion polymorphism in these species. The many different methodologies required to resolve these biologically important genetic variants highlight the challenges posed by SV in comparative primate genomics.

**Figure 1:**
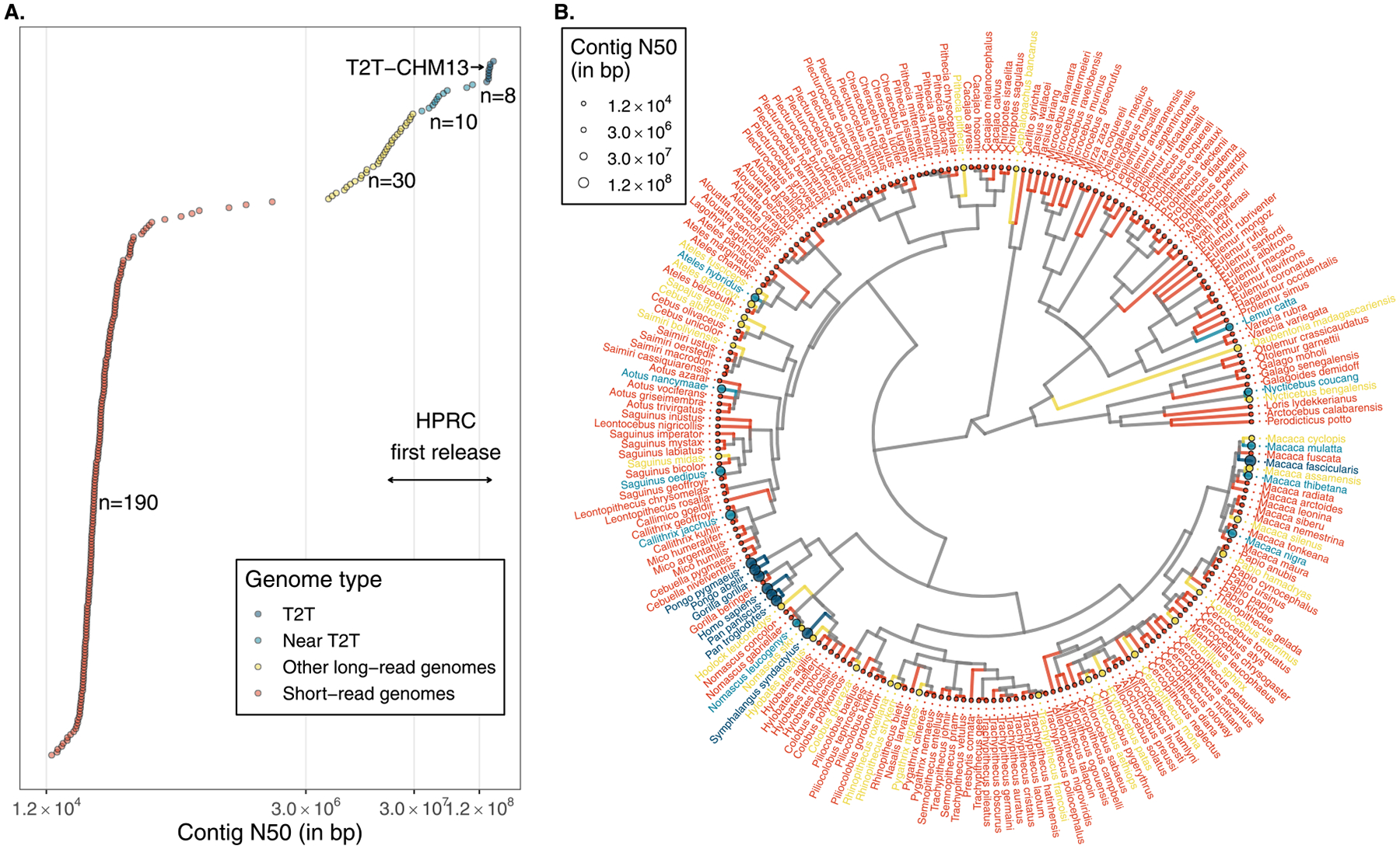
The current state of primate genome assemblies. **(a)** Contig N50 values of 238 primate species that have one or more genome assemblies available on National Center for Biotechnology Information (out of a total of > 500 recognized species). When multiple assemblies are available for the same species, the highest N50 value is shown, except for in the case of humans where the N50 of T2T-CHM13 is shown. Colors correspond to the type and quality of genome assemblies. The range of contig N50 values of the first draft human pangenome reference release by the HPRC is indicated by the double-headed arrow. **(b)** A phylogeny of these primate species obtained from TimeTree5 [71] excluding 16 species with no data available. Tips of the tree are colored according to **(a)**, and point sizes correspond to contig N50 values.

The recent advent of improved PacBio sequencing (HiFi) in combination with UltraLong Oxford Nanopore sequencing has vastly streamlined the process of primate genome assembly. Using these methods, Mao et al. [13] recently assembled the genomes of eight nonhuman primate species spanning ~50 million years of primate evolution (chimpanzee, bonobo, gorilla, orangutan, gibbon, macaque, owl monkey, and marmoset; Figure 1a,b). This deep survey of the primate phylogeny reveals that over this period ~27% of the primate genome has been impacted by structural variation. Furthermore, these genomes enabled the first complete characterization of > 1600 large structurally divergent regions (SDRs), defined as blocks of sequence ≥10 kb long that are highly divergent (≤85% identical). Strikingly, more than one-third of these SDRs exhibited recurrent structural variation intersecting 631 genes. Many of these genes that have been repeatedly gained or lost have medically relevant functions (e.g. RanBP2-like, GRIP domain-containing proteins, amylase (AMY), major histocompatibility complex [MHC]), highlighting loci potentially associated with adaptations. Thus, a complete understanding of primate evolution is achieved not just by more complete and contiguous genome assemblies but also by ensuring the representation of diverse taxa. Notable efforts have been made toward broadening taxonomic representation in primate comparative genomics with the release of high-coverage Illumina short-read genomes for 233 primate species [14–16], though insights into the SV diversity of these taxa will require comprehensive long-read sequencing and assembly efforts (Figure 1a,b).

## Population-level characterization of structural variation in humans in the pre-telomere-to-telomere era

The long-read sequencing frameworks used to identify lineage-specific SVs across primate evolution have also been exceptionally powerful in characterizing SVs segregating across human populations. With just 15 long-read sequenced genomes aligned to the human reference genome (GRCh38), Audano et al. [17] were able to detect 99 604 SVs, the vast majority of which (87.3%) were uncharacterized despite previous short-read-based discovery efforts on thousands of samples (e.g. [18]). SVs were strongly enriched in subtelomeric regions that are often composed of variable number tandem repeats inaccessible to short-read sequencing. However, these early long-read-based SV discovery efforts were still constrained by a limited sample size as well as the technology and reference genome available at the time. For example, large SD blocks and inversions remained inaccessible due to their complex nature. Moreover, as revealed by Ref. [17] GRCh38 contains biases and errors, in addition to large gaps in centromeric and acrocentric regions. The vast amount of SVs discovered suggests that a single linear reference genome cannot fully capture the content and variation commonly found in human genomes.

These findings directed subsequent efforts toward (1) more diverse representation of human populations, (2) discovery and genotyping of previously inaccessible SV types, (3) constructing a telomere-to-telomere (T2T) reference genome, and (4) employing pangenome graph–based references. Working toward increasing the representation of human diversity, the Human Genome Structural Variation Consortium (HGSVC) sequenced and assembled 64 phased haplotypes (32 diploid genomes) from 26 different human populations using a combination of PacBio CLR, HiFi, and Strand-seq [19]. Owing to the diverse sampling approach, 42% of these SVs were found to be novel compared with previous long-read sequencing efforts. Further constructing pangenome graphs from these assemblies enabled fast and accurate population-scale SV genotyping from short reads using a k-mer-based approach [20] (see also Ref. [21]), and the discovery of 1526 SV eQTLs that impact gene expression. Many of these affected genes have been associated with important human phenotypes, such as a 1069 bp deletion linked to increased expression of *LIPI*, which is associated with cardiac failure [22]. Additionally, 117 loci were identified exhibiting population stratification potentially playing roles in local adaptation and evolutionary tradeoffs. These include a 2.8 kbp insertion in an intron of CLEC16A, the disruption of which has been linked to type 1 diabetes. Interestingly, the frequency of this insertion is highest in Peruvians, a population with one of the highest type 1 diabetes rates [23].

To characterize the diversity of inversions in humans inaccessible to long reads alone, Porubsky et al. used Strand-seq to profile 41 human genomes [24] cataloging 729 inversions genome wide. Forty of these inversions were found to be recurrent with mutations occurring at rates up to 27 000-fold that of the average single-nucleotide variant mutation rate. These frequently ‘*toggling*’ inversions tend to be found in regions associated with genomic disorders, highlighting their potential roles in disrupting genomic stability and promoting deleterious SVs. These results mirror the recurrence of inversions observed in comparative studies among primates and highlight the distinct evolutionary processes unique to SVs.

## A new era of structural variants born from telomere-to-telomere and pangenomes

### Accessing structurally complex regions of human and nonhuman primate genomes with telomere-to-telomere assemblies

**Figure 2:**
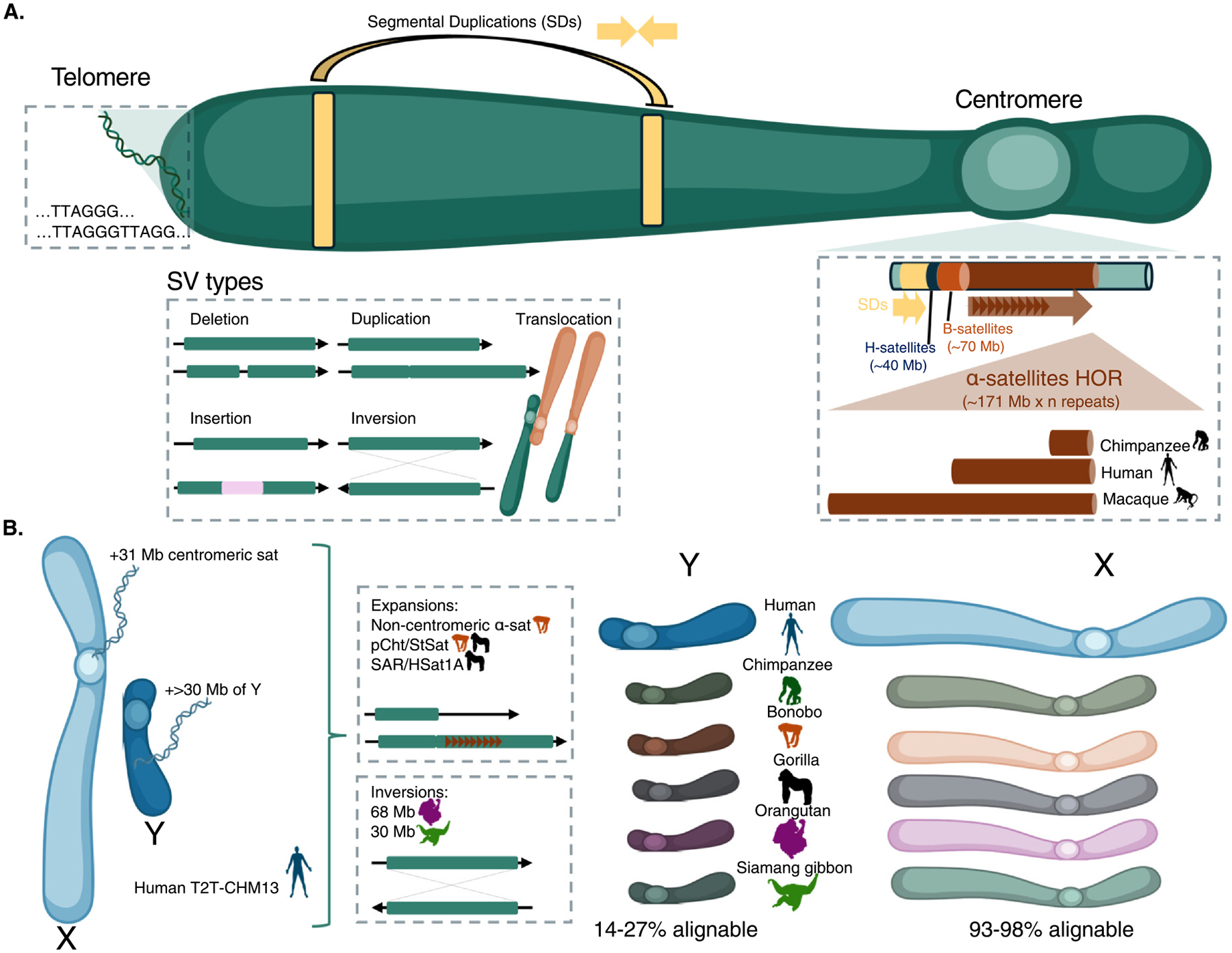
Key insights from the first complete genome assembly. **(a)** Schematic representation of the first T2T human genome assembly (T2T-CHM13) highlighting complete resolution of the sequence and structure of human telomeres and centromeres, as well as several different types of structural variation that can now be fully dissected and investigated in the context of haplotypes on which they emerged. A zoom in on centromeric regions illustrates the diversity of SVs and differences in centromeric length between humans and other great apes. **(b)** Illustration of the insights gained from the complete sequence and comparative analysis of human and great ape sex chromosomes. Among the newly added regions of the genome in T2T-CHM13 (> 82 Mb of unique sequence added) are 31 Mb of additional centromeric sequence at the X chromosome and over 30 Mb of Y chromosome sequence. The alignment of great ape sex chromosomes to human T2T-CHM13 further reveals extensive and complex structural diversity across the primate Y chromosome. Species silhouettes are from phylopic.org, and chromosome silhouettes created with BioRender.com.

The publication of the T2T human genome (T2T-CHM13) marked an end to more than 20 years of efforts to sequence and assemble a human genome from end-to-end [25] and a new beginning for comparative and population genomics (Figure 1a,b). This achievement unlocked access to several previously intractable regions of the genome including rDNA repeats and acrocentric DNA, SDs, centromeres, variable number tandem repeats, and telomeres (Figure 2a). These regions have been challenging to sequence and assemble for several different reasons (see for example, the importance of non-bDNA [26]), yet they all exhibit extensive structural variation both within and between species. T2T genome assemblies now enable us to reliably discover and genotype this SV for the first time.

Human centromeres, which are some of the most rapidly evolving regions in the genome, perfectly illustrate the power of T2T genomes. These centromeres are composed of tandemly repeated blocks of α-satellite each ~171 bp long, which are in turn organized into higher order repeats (HORs) and flanked by other complex satellite repeat sequences. These sequences differ remarkably both within and between species [27,28], yet are only now able to be sequenced and assembled. Comparing T2T-CHM13 to a newly sequenced near-T2T reference genome CHM1, Logsdon et al. recently performed the first genome-wide assessment of human centromeric structural variation [29]. This work un-covered extreme SNV diversity and size differences between the two sets of centromeres and their respective flanks (4.1-fold and threefold higher, respectively) with most hotspots of accelerated SNVs and SVs occurring within the core of the centromeric α-satellite HOR arrays (Figure 2a). Additional comparisons of the sequence and structure of six sets of centromeres from a chimpanzee, orangutan, and macaque showcased remarkably complex evolutionary dynamics, with α-satellite HORs almost completely unique in each species. Chimpanzee α-satellite HOR arrays were substantially smaller than humans, while macaque arrays were the largest among the primates assessed, though less complex and highly similar between homologs. In contrast, orangutan α-satellite HORs were highly divergent between homologs. These insights represent just the first glimpse of diversity at primate centromeres, which remain largely unexplored.

Sex chromosomes exhibit unique evolutionary signatures and structures in contrast to autosomes [30,31]. The primate X and Y chromosomes evolved from an autosomal pair ~170 MYA [32] and still contain *ancestral regions* derived from their ancestral autosomes, but only recombine across a shared *pseudoautosomal region* (PAR). The Y (and to a much lesser extent the X) also harbors *ampliconic sequences*, long (> 90 kbp) blocks of multicopy sequence, embedded in which are *palindromic sequences*, long inverted duplications that exhibit > 99.8% identity maintained by interlocus gene conversion [33,34]. The human X [35] and Y [36] chromosomes were recently sequenced T2T illuminating a previously unsequenced 3.1 Mb of centromeric satellite sequence on the X and adding > 30 Mb of new sequence to the Y chromosome assembly, including the massive q-arm DYZ1/DYZ2 heterochromatic repeats and the composite repeat structures of the *TSPY, RBMY*, and *DAZ* ampliconic gene families (Figure 2b).

Because the Y chromosome no longer recombines outside of the PAR, it has undergone extraordinary structural changes through evolution. A recent comparison of primate karyotypes showed greater than sixfold variation in Y chromosome size over the last ~70–80 MYA [37]. Recently, employing the same T2T assembly approaches as the human sex chromosomes, Makova et al. generated complete assemblies of the X and Y in five great apes (chimpanzee, bonobo, gorilla, Bornean, and Sumatran orangutan) and a siamang gibbon [38]. This effort found that while 93–98% of the human X chromosome was alignable across these primates, only 14–27% of the Y chromosome can be aligned, highlighting its exceptional evolutionary turnover (Figure 2b). Y chromosomes were enriched for inversions and insertions compared with the X and ranged from 30 Mb in Siamang to 68 Mb in Sumatran Orangutan, with ampliconic sequences driving these size variations. Multicopy ampliconic genes on the human Y chromosome are associated with spermatogenesis. However, several species show lineage-specific expansions in these ampliconic genes, which tend to lie in palindromes, suggesting potential interspecific differences in the molecular underpinnings of reproduction. Several species also exhibit unique satellite sequence expansions on the X and Y, including extensive subtelomeric pCht/StSat expansion in gorillas, SAR/HSat1A sequences in bonobos and gorillas, and noncentromeric α-satellites in bonobos. Together, these analyses highlight the important role of SVs in primate sex chromosome evolution and the novel insights that can be gained in the era of T2T comparative genomics.

In addition to comparative studies of T2T assemblies, T2T genomes are powerful resources for population-level studies of structural variation when serving as a single linear reference. By aligning long reads from 17 diverse samples to different iterations of the human reference, Aganezov et al. [39] demonstrated that the errors and biases in GRCh38 previously documented by Ref. [17] are much reduced in T2T-CHM13. This results in more accurate SV calls and a more balanced insertion-to-deletion ratio. In addition, SV discovery is made possible at many structurally complex regions that were previously unresolved, although highly repetitive regions still pose a challenge due to poor alignment rates. Furthermore, Porubsky et al. [40] remapped the Strand-seq data generated from Ref. [24] to T2T-CHM13 and showed that the T2T assembly increases inversion discovery by ~21%. Using 10 additional Strand-seq samples, they were able to identify five more novel rare inversions, four of which overlap with CNVs previously associated with disease (e.g. autism, Cooper syndrome, and prostate cancer). Interestingly, comparing these to other primates, they found that the minor allele represents the ancestral orientation in three cases out of five.

### Unlocking complex structural diversity with human pangenomes

Despite having laid the foundation for SV discovery between and within primate species, T2T references are still single, haploid reference structures that cannot fully represent many common variants [41,42]. The publication of the first draft of the human pangenome by the Human Pangenome Reference Consortium (HPRC) popularized the concept of a multigenome graph-based reference, making it possible to accurately represent, phase, and assemble up to 99% of the genome of any individual (Figure 1a) [43]. With an initial set of 47 phased diploid genomes from individuals representing genetically diverse populations worldwide, this pangenome reference was able to represent most of the common genetic variation in humans. Genotyping short-read data with this pangenome reference [20] further illustrate its utility in the study of SVs with 104% more SVs identified per haplotype compared with mapping the same data to a single linear reference. In addition, when compared with the pangenome graph generated by HGSVC [19], the HPRC pangenome was able to detect 34.8% more SVs per haplotype. These improvements reflect the enhanced representation of structurally complex regions in the HPRC pangenome. More importantly, this graph-based population-scale effort has greatly added to our understanding of the changes in genome structure and mutational processes that give rise to human genetic diversity.

For instance, while T2T-CHM13 helped resolve the sequence and structure of the human acrocentric chromosomes, the underlying mechanisms leading to the observed patterns of homology remained unclear. The first human pangenome was therefore key to identifying ongoing recombination between heterologous acrocentric chromosomes, solidifying a 50-year-old cytogenetics-based hypothesis for the origins of recurrent Robertosonian translocations [44]. Similarly, T2T-CHM13 enabled the discovery of > 51 Mb of previously unresolved SDs and lineage-specific gene duplications [45]. Exploring this sequence in the context of 102 additional phased human haplotypes, however, revealed distinct patterns of evolution and mutation [46]. Among these is the discovery that SDs harbor 60% more diversity than unique regions of the genome, 27% of which arises through interlocus gene conversion. A pangenomic interrogation of 43 Y chromosome assemblies revealed extensive structural diversity among humans of different ancestry [47], extending on the observations of the T2T Y [36]. Similar to interspecies comparisons of Y chromosomes, human Y chromosomes varied vastly in size (45.2–84.9 Mb) with heterochromatic sequence contributing most prolifically. In contrast, the euchromatic sequence exhibited little variation in size but was susceptible to recurrent large inversions, occurring at a rate greater than twofold higher than the rest of the genome. Finally, recent efforts to appraise telomere length variation across a diversity panel of 147 human individuals [48] revealed that different chromosomes exhibit conserved, distinct lengths. Nevertheless, telomere length differed extensively (> 6 Kb) among individuals. Telomeres are known to shorten throughout lifespan and shortened telomere length has been associated with several age-related diseases [49], while longer telomere lengths have been associated with cancer [50]. Differences and conservation in telomere lengths across primate chromosomes remain completely unexplored however. Together, these results reveal the cutting edge in probing SV across the complex genome at the population level.

## Future directions and outlook

**Figure 3:**
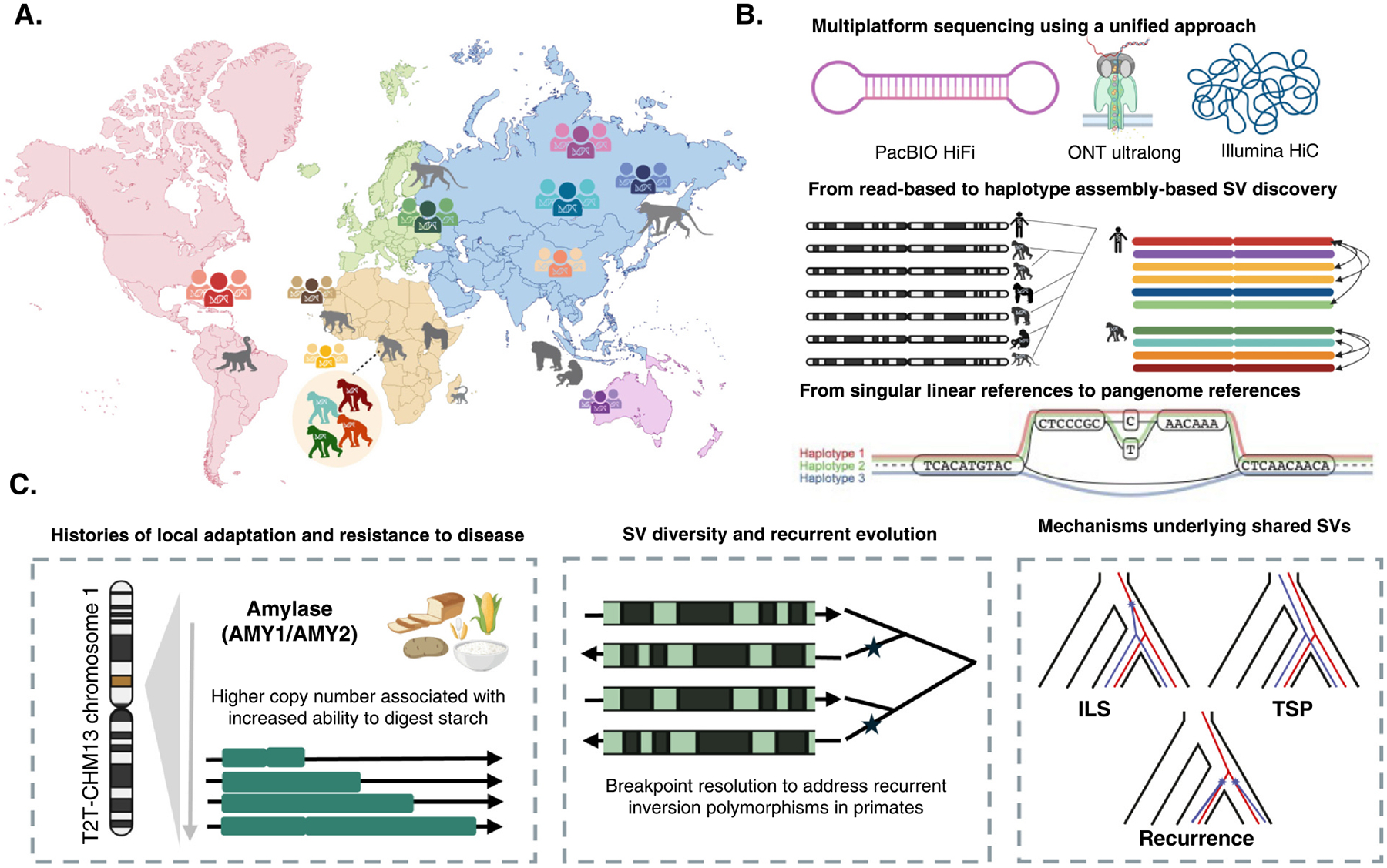
Opportunities and outlook for future research investigating primate structural diversity in the era of T2T and pangenomes. **(a)** Ongoing and future efforts to generate additional T2T reference genomes and population-scale haplotype-resolved assemblies for humans and nonhuman primates. **(b)** The momentum created by advances in sequencing technology and assembly algorithms shifted the paradigm for SV discovery from read-based SV discovery to haplotype-resolved assembly-based SV discovery, as well as from singular references to pangenomes. **(c)** A subset of research areas on the origins, diversity, and evolutionary history of complex SVs that can now be fully investigated within and between species: (1) association with local adaptation and resistance to disease (here exemplified by selection on increased copy number at the amylase locus amidst the rise of agriculture); (2) recurrent evolution in primates (here exemplified by recurrent inversion polymorphisms); and (3) mechanisms underlying structural diversity that is shared between humans and our primate kin. Created with BioRender.com.

The study of structural variation is undergoing a revolution driven by new sequencing technologies that have enabled us to peer deep into complex regions of the genome with unprecedented resolution. Consortia such as the HPRC and HGSVC have already transformed our understanding of human SV, and these projects are poised to catalog the diversity of humans worldwide over the next several years (Figure 3a). Nevertheless, substantial additional effort will be required to further enhance the quality of long-read genome assemblies [51] and to fully capture the extensive diversity driving locally adaptive signatures in distinct human populations. Additionally, there is a pressing need for similar efforts in nonhuman primates both to contrast with human diversity and understand the genetic basis of what makes us human. Several T2T genomes of apes are indeed expected in the near future alongside a T2T crab-eating macaque (*Macaca fascicularis*; Figures 1a,b and 3a) [52]. Yet, there are more than 500 recognized primate species [53], and thus, a vast task remains ahead in probing the T2T diversity of this clade spanning ~70–80 million years. As evidenced by the extensive insights gained from human pangenomics, nonhuman primate multi-individual pangenome efforts are essential to understanding the evolution and diversity of these species and gaining insights into loci driving speciation and adaptation (Figure 3b,c), which can inform conservation efforts. Although considerable challenges still remain in applying pangenomic methods for the study of SV evolution in nonhuman species and in interspecific comparisons (e.g. higher levels of divergence and structural complexity [54,55], and uneven quality in genome annotations [13]), they also represent numerous exciting opportunities to be explored.

The influx of haplotype-resolved human genomes has already enabled the revisiting of several loci with intriguing relevance to health and human evolution [43,56,57]. These include the amylase locus, which encodes for several proteins that facilitate the digestion of starches in saliva and the pancreas [58]. While previous work had hinted at the complexity of genes and their adaptive potential [59], recent exploration of long-read assemblies has resolved the complete diversity of this locus for the first time and identified previously unknown selective signatures (Figure 3c). Similarly, these same long-read assemblies enabled the discovery of three distinct structural haplotypes at the KLRC2 locus, a gene involved in immune cell maturation and the deletion of which has been associated with severe COVID-19 symptoms [60]. These haplotypes harbor 0, 1, and 2 copies of the KLRC2 gene, and they were shown to have arisen from one deletion event and one duplication event. Nevertheless, the human genome is home to hundreds of such complex regions (see Ref. [61] for a review of several loci of interest). Some loci such as the defensins, which play a role in the innate immune system, exhibit extraordinary variation among humans [61,62] and yet were not even fully assembled in the human reference genome before T2T-CHM13 [28]. Dissecting the complex evolution of each of these loci over the next several years will unveil exciting insights into human adaptation and diversity. Furthermore, these loci have been largely invisible to association studies and yet are likely in many cases to play roles in human diseases and other phenotypes.

One of the major insights from recent profiling of inversions both within and between species [11] as well as studies of individual loci such as amylase [56] is the extensive recurrence of SV. This is in stark contrast to single base substitutions where homoplasy is extremely rare. Yet, for several forms of SV, similar and identical structures can emerge independently. This is in part due to the exceptionally high mutation rates at these loci, which can be as much as four orders of magnitude higher than the estimated single base pair substitution mutation rates [24,56]. Evolutionary analyses of such loci thus must appraise these exceptional mutational properties, which in many cases can invalidate the assumptions behind population genetic theory or methods, to identify haplotypes associated with disease (Figure 3c).

Recent primate sequencing efforts have highlighted extensive gene tree versus species tree discordances, often caused by incomplete lineage sorting (ILS) and also impacted by selection [63,64]. T2T primate genomes can be used to revisit primate ILS topologies and, critically, to interrogate SVs and diverse haplotypes affected by ILS and potentially targeted by selection. Leveraging complete haplotype-resolved assemblies from primate populations will further enable distinguishing SVs and complex haplotypes that evolved independently (i.e. recurrent/identical-by-state), from *trans-species polymorphisms* (TSPs) maintained by balancing selection [65]. While presumed rare in the human genome, TSP loci have been historically difficult to identify as they harbor deeply divergent alleles poorly captured by short-read sequencing [66]. For instance, the MHC locus, a textbook example of TSP, was recently shown to have alleles shared as distantly as between humans and new-world monkeys [67], yet its sequence and structure remain largely unresolved both within and between primate species. Emerging research is now providing increasing evidence of SVs maintained by balancing selection, including extensive multiallelic copy number variation at the salivary agglutinins gene (DMBT1) [68], and trans-species deletion polymorphisms affecting genes involved in the immune system and metabolism [69]. Future efforts to generate human and primate pangenomes are likely to expand our understanding of key loci of interest, such as MHC, and identify as-yet undiscovered TSPs among diverse species.

The field of genomics is driven by technological and methodological advances enabling novel discovery as well as reappraisals of known patterns of diversity. Long-read sequencing is now providing an unprecedented view of structural variation in primate genomes. These advances have unlocked a new era with opportunities for novel synthesis across different disciplines to gain deeper biological insights into the inter- and intra-species diversity, mutational mechanisms, evolutionary histories, and functional impact of these complex regions of the genome.

## Data Availability

No data were used for the research described in the article.

## Glossary

Acrocentric chromosome: A chromosome where the centromere is located near an end of the chromosome. The short arms of human acrocentric chromosomes 13, 14, 15, 21, and 22 harbor ribosomal DNA (rDNA) repeats and large segmental duplications (SDs)
Ampliconic sequences: One of the three classes of male-specific euchromatic sequences of primate Y chromosomes. They are highly repetitive and harbor multicopy gene families that have key functions related to reproduction in males
Centromere: The region of the genome that links a pair of sister chromatids together during cell division. Primate centromeres are mostly composed of α-satellite repeats, which are in turn organized into HORs
CHM1: A complete hydatidiform mole (CHM) cell line for which a nearly T2T reference genome is available
Euchromatin: Lightly packed chromatin that is more access to transcription and is enriched in genes
Expression quantitative trait locus (eQTL): A locus that is associated with the expression level of a gene
Genome Reference Consortium Human Build 38 (GRCh38): The most recent build of the human reference genome released by the Genome Reference Consortium before T2T-CHM13. It is a linear composite of merged haplotypes from more than 20 people, with a single individual contributing ~70% of the sequence [70]
Heterochromatin: Tightly packed chromatin that is often inaccessible to transcription and composed of repetitive sequences
Higher-order repeat (HOR): A series of copies of larger repeat units, each of which consisting of multiple smaller repeat units
Homoplasy: A shared feature that has evolved independently multiple times. This is in contrast to homology, where a shared feature can be explained by a common origin
Human Genome Structural Variation Consortium (HGSVC): A project aimed to reveal the full spectrum of human genetic variation, with a focus on structural variation
Human Pangenome Reference Consortium (HPRC): A project aimed to sequence and assemble genomes from individuals from diverse populations in order to better represent the genomic landscape of diverse human populations. The first draft pangenome from HRPC consists of 47 haplotype-resolved human references chosen from the 1000 Genomes Project diversity panel, with ongoing efforts to sequence more than 500 diverse human samples worldwide
Incomplete lineage sorting (ILS): The failure for lineages within a population to coalesce before reaching the common ancestral population with another population. It leads to discordance between gene trees and the species tree, which includes trans-species polymorphisms. Other causes of such incongruences include introgression and recurrent mutation. ILS can occur by neutral processes alone, or can be facilitated by selection
Nonallelic homologous recombination (NAHR): Crossing-over between paralogous sequences. This mutational process can lead to structural variation (e.g. deletions, duplications, and inversions)
Pangenome reference: A reference genome for a given species consisting of multiple genomes often organized in a graph format that is aimed to represent all the common variation present in that species
Polymorphism: The occurrence of two or more genotypes at a given locus in a population. **Pseudoautosomal region (PAR)**: Homologous sequences on the X and Y chromosomes that are derived from a common ancestor
Robertosonian translocation: A form of translocation where two acrocentric chromosomes break at their centromeric regions and their long arms are fused together
Segmental duplication (SD): DNA sequences ≥1 kbp that are duplicated in a genome with ≥90% sequence identity to each other
Single nucleotide variant (SNV): A substitution of a single nucleotide at a certain position in the genome
Structurally divergent region (SDR): Large regions of the genome, which are highly divergent and often impacted by multiple complex SV events (≥10 kbp in length and ≤85% in similarity)
Structural variant (SV): A genetic variant altering the structure of a genome typically longer than 50 bp. It consists of insertions, deletions, inversions, duplication, and translocations
T2T-CHM13: The first complete and gapless telomere-to-telomere (T2T) human reference genome released by the T2T Consortium. This genome is derived from a complete hydatidiform mole (CHM) cell line that is almost completely homozygous
Tandem repeat: A sequence of two or more base pairs that is repeated multiple times adjacent to each other
Telomere-to-telomere (T2T) genome assembly: An assembly that covers the whole of each chromosome without gaps and is free from large-scale assembly errors
Trans-species polymorphisms (TSP): Ancient genetic variation, whose origin predates speciation events, that remains segregated in each species. TSP in highly diverged species in highly diverged species can often be a sign of balancing selection
